# Zufriedenheit von Patienten und Ärzten mit Videokonsultationen in der Dermatologie: Eine explorative Querschnittsstudie

**DOI:** 10.1111/ddg.70035x

**Published:** 2026-10-05

**Authors:** Felix von Krogh, Julia Zarnowski, Regina Treudler, Jan‐Christoph Simon, Anna‐Theresa Seitz

**Affiliations:** ^1^ Klinik und Poliklinik für Dermatologie Venerologie und Allergologie Universitätsklinikum Leipzig AöR Leipzig Deutschland; ^2^ Institut für Allergieforschung Charité – Universitätsmedizin Berlin Corporate Member of Freie Universität Berlin and Humboldt‐Universität zu Berlin Berlin Deutschland

**Keywords:** Barrieren, Beeinträchtigungen, Beurteilung von Hautbefunden, Teledermatologie, Verbindungsqualität, Videosprechstunde, Zufriedenheit, assessment of skin findings, barriers, connection quality, impairment, satisfaction, teledermatology, video consultation

## Abstract

**Hintergrund und Ziele**: Die Teledermatologie stellt einen zunehmend wichtigen Bestandteil der dermatologischen Versorgung dar, ist im klinischen Alltag jedoch weiterhin mit Herausforderungen bei der Implementierung konfrontiert. Im Zeitraum von einem Jahr wurde eine Befragung zu Videokonsultationen durchgeführt, um die Zufriedenheit sowie Implementationsbarrieren bei Patienten und Ärzten zu evaluieren.

**Patienten und Methodik**: In einer monozentrischen, fragebogenbasierten Studie wurde die Zufriedenheit von Patienten und Ärzten erfasst. Hierzu wurden ein eigens entwickelter Fragebogen sowie die deutsche Adaptation des validierten Client Satisfaction Questionnaire eingesetzt.

**Ergebnisse**: Von 250 geplanten Konsultationen fanden 89,6% erfolgreich statt. Insgesamt nahmen 128 Patienten und 13 Ärzte an der Befragung teil. Die Mehrheit der Patienten (94,5%) und Ärzte (94,7%) würde Videokonsultationen weiterempfehlen. 93% der Patienten gaben positives Feedback zur Benutzerfreundlichkeit und 80,4% zur Gesamterfahrung. 98% der Ärzte gaben positives Feedback zur Terminplanung, 94,7% zur Benutzerfreundlichkeit und 90,8% zur GesamterfahrungDie Bewertung der Verbindungsstabilität und der Qualität der Hautbefundung fiel heterogen aus. Ärzte, die mit der Hautbefundung unzufrieden waren, empfahlen Videokonsultationen seltener (*p* < 0,001) und berichteten über eine schlechtere Gesamterfahrung (*p* < 0,001).

**Schlussfolgerungen**: Videokonsultationen können den Zugang zur dermatologischen Versorgung verbessern und zu einer effizienteren Ressourcennutzung beitragen. Unsere Studie zeigt eine hohe Akzeptanz bei Patienten und Ärzten, identifiziert jedoch auch Optimierungsbedarf, insbesondere im Hinblick auf die Videoqualität und die Zuverlässigkeit der Hautbefundung.

## EINLEITUNG

Die Teledermatologie hat sich rasch zu einem wesentlichen Bestandteil der dermatologischen Versorgung entwickelt, insbesondere aufgrund des technologischen Fortschritts und der wachsenden Nachfrage nach dermatologischen Dienstleistungen.^1^ Der Einsatz der Teledermatologie hat im Laufe der Jahre weltweit zugenommen, wobei sowohl in staatlich geförderten und privaten Gesundheitssystemen als auch in privaten Praxen ein deutlicher Anstieg zu beobachten ist.^2^, ^3^

Telekommunikationstechnologien ermöglichen Fernkonsultationen zwischen Patienten und Dermatologen, wodurch geografische Barrieren effektiv überwunden werden können und die Effizienz der dermatologischen Versorgung verbessert werden kann. Die COVID‐19‐Pandemie markierte einen Wendepunkt in der Teledermatologie. So wurde die dermatologische Versorgung in Europa durch die Pandemie stark beeinträchtigt, was sich in einem starken Rückgang der Patientenzahlen widerspiegelte. Beispielsweise gingen die dermatologischen Notfallkonsultationen am *Institute for Research, Hospitalization and Health Care in San Matteo*, Italien, um 79,9% zurück; von 164 im Jahr 2019 auf 33 im Jahr 2020.^4^ In ähnlicher Weise sanken die Krankenhausaufenthalte in der universitären dermatologischen Abteilung in Breslau, Polen, im gleichen Zeitraum um 71,9%, von 626 auf 176.^5^

In Bezug auf die dermatologische Versorgung in Deutschland führte das erste Jahr der Pandemie zu einem Rückgang der stationären Hauttumorbehandlungen um 14%; darunter fand sich ein Rückgang der Fallzahlen um 16% bei nichtmelanozytärem Hautkrebs und um 7% beim malignen Melanom.^6^ Darüber hinaus meldeten große Allergiezentren in deutschsprachigen Ländern einen Rückgang von fast 50% bei der Einleitung einer potenziell lebensrettenden spezifischen Immuntherapie für Patienten mit Insektengiftallergie.^7^ Demgegenüber beschleunigte die COVID‐19‐Pandemie die Einführung der Teledermatologie, da Gesundheitsdienstleister bestrebt waren, persönliche Konsultationen zu reduzieren und dennoch eine kontinuierliche Versorgung sicherzustellen.^8^, ^9^

Dennoch variiert die Verbreitung teledermatologischer Dienstleistungen erheblich und die Situation in Deutschland ‐ als europäisches Beispiel – ist nach wie vor suboptimal. Im Jahr 2021 gaben nur 7,6% der befragten Dermatologen an, Echtzeit‐Videokonsultationen mit Patienten anzubieten.^10^ Im Gegensatz dazu nutzten 42,6% der privaten Praxen und 30% der akademischen Einrichtungen in den Vereinigten Staaten vergleichbare Videoplattformen.^11^ Dieser erhebliche Unterschied unterstreicht den Bedarf für Verbesserungen in der Anwendung von Teledermatologie.

Mögliche Barrieren für die Implementierung teledermatologischer Anwendungen könnten in der begrenzten Akzeptanz bei Ärzten und Patienten, Schwierigkeiten im Umgang mit der Technologie, Problemen hinsichtlich der Qualität und Zuverlässigkeit von Videokonsultationen sowie Unklarheiten bei der Kostenerstattung liegen. Um diese Herausforderungen besser zu verstehen, führten wir von Januar bis Dezember 2021 eine Umfrage durch, um die Zufriedenheit von Patienten und Ärzten mit Videokonsultationen in der realen Versorgung zu bewerten. In dieser Studie bezieht sich der Begriff *tägliche Praxis* auf den routinemäßigen klinischen Einsatz von Videokonsultationen als Teil der Standardpatientenversorgung, unabhängig vom Studiendesign. Videokonsultationen wurden in unserer Einrichtung im vierten Quartal 2020 eingeführt und sind seitdem in den regulären ambulanten Arbeitsablauf integriert worden. Die Gewinnung neuer Erkenntnisse auf diesem Gebiet ist für die Optimierung digitaler Gesundheitsdienste und die Überwindung bestehender Hindernisse von entscheidender Bedeutung.

## MATERIAL UND METHODIK

### Studiendesign

Wir untersuchten die Zufriedenheit von Patienten und Ärzten und identifizierten gleichzeitig potenzielle Hindernisse innerhalb eines einjährigen Zeitraums im Jahr 2021 unter Verwendung eines monozentrischen, querschnittsorientierten, fragebogenbasierten und datengestützten Ansatzes. Patienten und Ärzte, die im Zeitraum von Januar 2021 bis Dezember 2021 an einer Live‐Videosprechstunde in den Abteilungen für Allergologie, Allgemeine Dermatologie, Andrologie und Lasermedizin der Klinik und Poliklinik für Dermatologie, Venerologie und Allergologie des Universitätsklinikums Leipzig teilgenommen hatten, wurden nach schriftlicher Einwilligungserklärung in die Studie eingeschlossen. Die Zufriedenheit von Patienten und Ärzten wurde nach erfolgreich durchgeführter Videosprechstunde mittels Fragebogen erfasst. Zusätzlich wurden Informationen zur Verbindungsqualität und ‐stabilität, zum verwendeten Gerät und Betriebssystem, Alter, Geschlecht, Beschäftigungsstatus und zur jeweiligen Abteilung erhoben. Potenzielle Einflussfaktoren wie Computerkenntnisse und der Bedarf an technischer Unterstützung wurden berücksichtigt. Es wurden keine formalen Effektmodifikatoren definiert, jedoch untersuchten wir Gerätetyp und Abteilung als mögliche Einflussfaktoren. Zur Verringerung potenzieller Verzerrungen wurden alle Teilnehmer eingeladen und standardisierte Fragebögen verwendet. Wenn Patienten oder Ärzte den Fragebogen nicht ausfüllten, wurden ihre Perspektiven zur Zufriedenheit nicht in die Zufriedenheitsanalyse einbezogen. Dieses Manuskript wurde gemäß den STROBE‐Empfehlungen (Strengthening the Reporting of Observational Studies in Epidemiology) erstellt.^12^

### Ethik und Datenschutz

Die Studie wurde in Übereinstimmung mit der Deklaration von Helsinki durchgeführt und von der lokalen Ethikkommission (Medizinischen Fakultät der Universität Leipzig (Ethikvotum 029/21‐ek) genehmigt. Alle Teilnehmer gaben nach Aufklärung ihre schriftliche Einwilligung vor der Studienteilnahme. Die Videosprechkonsultationen wurden mithilfe der zertifizierten E‐Health‐Software Samedi durchgeführt, die den Anforderungen der europäischen Datenschutz‐Grundverordnung (DSGVO) entspricht. Wir nutzten die Funktionen von Samedi zur Terminplanung und Durchführung der Videokonsultationen. Die Samedi GmbH, die diese Plattform bereitstellt, fungiert als Auftragsverarbeiter im Auftrag der Gesundheitseinrichtung, die als Verantwortlicher im Sinne der Datenschutz‐Grundverordnung agiert. Laut der Datenschutzrichtlinie von Samedi wurden alle personenbezogenen und gesundheitsbezogenen Daten vor der Übertragung lokal auf den Endgeräten der Nutzer verschlüsselt, sodass unverschlüsselte Daten nicht auf den Servern von Samedi zugänglich sind. Für die Datenerhebung erhielten alle teilnehmenden Patienten und Ärzte den Fragebogen entweder postalisch oder per verschlüsselter E‐Mail über den Klinikserver zugestellt. Alle Antworten wurden in Excel‐Tabellen zusammengeführt und auf Klinikrechnern sowie internen Servern gesichert, sodass sämtliche Daten innerhalb des geschützten Netzwerks der Einrichtung verblieben. Es wurden keine Cloud‐basierten oder externen Speicherdienste genutzt. Alle Verfahren entsprachen den geltenden Datenschutzgesetzen und den institutsinternen Richtlinien zur Gewährleistung der Datensicherheit.

### Fragebögen

Die teilnehmenden Patienten und Ärzte erhielten innerhalb einer Woche nach erfolgreichem Videosprechstundentermin einen intern entwickelten Fragebogen per E‐Mail oder Post zugestellt. Der Fragebogen erfasste demografische Daten sowie eine Bewertung von Terminvergabe, Benutzerfreundlichkeit, Verbindungsstabilität und das Gesamterlebnis mithilfe des deutschen Schulnotensystems (1 = *sehr gut* bis 6 = *ungenügend*). Weitere Fragen bezogen sich auf die Weiterempfehlung der Videokonsultation an Freunde oder Familienangehörige (ja/nein), das verwendete Endgerät sowie Betriebssystem. Diese Fragen wurden aus einer zuvor veröffentlichten Studie übernommen, die die Zufriedenheit mit Videosprechstunden in einer ophthalmologischen Abteilung untersuchte.^13^

Darüber hinaus wurden die Patienten gebeten, die validierte deutsche Version des ursprünglich von Larsen et al. entwickelten *Client Satisfaction Questionnaire* (CSQ) auszufüllen, den *Fragebogen zur Messung der Patientenzufriedenheit (ZUF‐8*).^14^, ^15^ Dieser Fragebogen misst die Patientenzufriedenheit, indem die Qualität und Zufriedenheit mit der Behandlung, die Erfüllung der Patientenbedürfnisse und der Umgang mit Problemen bewertet werden. Der Fragebogen besteht aus acht Items, die jeweils von 1 bis 4 bewertet werden. Die Werte aller acht Items werden addiert und können einen Gesamtwert von 8 bis 32 erreichen. Höhere Punktwerte weisen auf eine höhere Zufriedenheit mit der Behandlung hin, während niedrige Werte eine geringere Zufriedenheit anzeigen. Das in dieser Studie verwendete Instrument zur Befragung von Ärzten wurde intern entwickelt, um die Erfahrungen der Nutzer bei teledermatologischen Konsultationen spezifisch und detailliert zu bewerten. Das Instrument wurde zwar nicht formal validiert, aber es wurde auf der Grundlage praktischer Überlegungen entwickelt, die für den Kontext der Teledermatologie relevant sind. Zur Bewertung der internen Konsistenz der Ärztebefragung mittels Cronbachs alpha wurde eine Reihe von fünf inhaltlich unterschiedlichen, jedoch konzeptionell zusammenhängenden Items herangezogen. Diese wurden von den Ärzten anhand derselben Bewertungsskala (deutsche Schulnoten) beurteilt und umfassten die Bereiche Terminplanung, Benutzerfreundlichkeit, Verbindungsstabilität, Gesamterfahrung sowie die Beurteilbarkeit der Hautbefunde. Die Analyse ergab einen Zuverlässigkeitskoeffizienten von α = 0,806, was auf eine gute Zuverlässigkeit hinweist. Das deutsche Schulnotensystem wurde bei Patienten und Ärzten eingesetzt, um eine intuitive Beantwortung der Fragen zu ermöglichen.

### Statistische Analyse

Die statistische Analyse wurde mit IBM SPSS Statistics, Version 24.0, durchgeführt. In dieser Studie wurde ein explorativer statistischer Ansatz gewählt, anstatt eine spezifische Hypothese zu prüfen. Angesichts des beobachtenden Charakters der Studie wurde keine formale Berechnung der Stichprobengröße oder eine A‐priori‐Poweranalyse durchgeführt. Die Stichprobengröße ergab sich aus der Zahl der im Studienzeitraum teilnahmeberechtigten und einwilligenden Patienten. Deskriptive Ergebnisse wurden als absolute und relative Häufigkeiten angegeben. Für kontinuierliche Variablen wurden Mittelwerte und Standardabweichungen berechnet, für ordinale Variablen Mediane und Interquartilsabstände (IQR). Die Normalverteilung wurde mit dem Shapiro‐Wilk‐Test überprüft. Da der Client Satisfaction Score (CSQ) keine Normalverteilung aufwies, kamen nichtparametrische Tests zur Anwendung. Der Zusammenhang zwischen kontinuierlichen Variablen (zum Beispiel Alter) wurde anhand der Spearman‐Rangkorrelation bewertet, Unterschiede in den Zufriedenheitswerten zwischen kategorialen Variabl en mit binären Gruppen (zum Beispiel Geschlecht) wurden mit dem Mann‐Whitney‐U‐Test geprüft, während Variablen mit mehr als zwei Gruppen (zum Beispiel Beschäftigungsstatus, Betriebssystem, Gerät, Abteilung) mit dem Kruskal‐Wallis‐Test analysiert wurden. Ein p‐Wert von < 0,05 wurde als statistisch signifikant angesehen.

## ERGEBNISSE

### Allgemeine Daten

Von Januar 2021 bis Dezember 2021 wurden insgesamt 250 Videokonsultationen terminiert. Die meisten Termine wurden im Februar vereinbart (n = 39; 15,6%), während die wenigsten im Oktober geplant waren (n = 10; 4%). In allen anderen Monaten wurden zwischen 17 und 24 Termine (6,8–9,6%) geplant. Die meisten Patienten hatten einen Termin für eine allergologische Konsultation (n = 135; 54%), gefolgt von einer Konsultation in der allgemeinen Dermatologie (n = 63; 25,2%) und in der Laserabteilung (n = 44; 17,6%). Die wenigsten Termine wurden in der Andrologieabteilung vereinbart (n = 8; 3,2%).

Von den 250 terminierten Videokonsultationen wurden 224 (89,6%) durchgeführt; die No‐Show‐Rate der Patienten lag bei 10,4%. Von den 224 durchgeführten Videokonsultationen wurden 206 (92%) mit voller Audio‐ und Videoqualität durchgeführt, während bei 8% aller Konsultationen eine Beeinträchtigung der Ton‐ oder Bildqualität berichtet wurde. Bei 13,2% (n = 33) aller Videokonsultationen wurde eine stationäre Aufnahme empfohlen, bei 33,6% (n = 84) wurde eine medikamentöse Therapie eingeleitet oder angepasst und in 13,6% (n = 34) der Fälle wurde ein Rezept ausgestellt.

Häufige Diagnosen, die im Rahmen der Videokonsultationen kodiert wurden, sind in Tabelle 1 aufgeführt. In der Allergologie waren der Verdacht auf Nahrungsmittelallergie (n = 22; 8,8%), Hymenopterengiftallergie (n = 16; 6,4%) und chronische Urtikaria (n = 16; 6,4%) die häufigsten Diagnosen, während in der allgemeinen Dermatologie Psoriasis (n = 9; 3,6%), melanozytäre Nävi (n = 8; 3,2%) und Rosazea (n = 7; 2,8%) am häufigsten vorkamen. Spidernävi (n = 5; 2,0%) und Hämangiome (n = 4; 1,6%) waren die häufigsten Diagnosen in der Laserabteilung.

**TABELLE 1:** Häufige Diagnosen im Rahmen der Videokonsultationen (von 250 geplanten Terminen).

Diagnose	ICD‐10	n	%
***Abteilung für Allergologie***			
Verdacht auf Nahrungsmittelallergie	T78.1	22	8,8
Hymenopterengiftallergie	T63.4	16	6,4
Chronische Urtikaria	L50.9	16	6,4
Verdacht auf Allergie gegen Analgetika	Z88.6	15	6,0
Allergische Rhinitis	J30.1	13	5,2
Verdacht auf Allergie gegen Impfstoffe	T88.1	12	4,8
Atopisches Ekzem	L20.8	7	2,8
Verdacht auf Allergie gegen Antibiotika	Z88.1	7	2,8
Typ‐IV‐Überempfindlichkeitsreaktion auf Arzneimittel	T88.7	6	2,4
Verdacht auf Allergie gegen Lokalanästhetika	Z88.4	4	1,6
Allergische Kontaktdermatitis durch Kosmetika	L23.2	3	1,2
***Abteilung für allgemeine Dermatologie***			
Psoriasis vulgaris	L40.0	9	3,6
Melanozytärer Nävus	D22.5	8	3,2
Rosazea	L71.8	7	2,8
Tinea unguium	B35.1	4	1,6
Lichen planopilaris	L43.8	3	1,2
Morphea	L94.0	3	1,2
***Laserabteilung***			
Spidernävus	I78.1	5	2,0
Hämangiom	D18.0	4	1,6
Xanthelasmen	H02.6	3	1,2
Hypertrichose	L68.9	3	1,2
Café‐au‐Lait‐Flecken	L81.3	3	1,2
Seborrhoische Keratose	L82	3	1,2

### Demografische Daten

Alle Patienten, die an der Videosprechstunde teilgenommen haben (n = 224) wurden per E‐Mail oder Post zur Teilnahme an der fragebogenbasierten Studie eingeladen, woraufhin 57,1% (128/224) antworteten. Die meisten teilnehmenden Patienten wurden in der allergologischen Abteilung konsultiert (n = 80/124; Rücklaufquote des Fragebogens 64,5%), gefolgt von der Laserabteilung (Patienten: 24/39; 61,5%) und der Abteilung für allgemeine Dermatologie (24/61; 39,3%). Kein Patient oder Arzt aus der andrologischen Abteilung hat auf die Studienanfrage geantwortet.

Alle 13 Ärzte, die regelmäßig Videokonsultationen durchführten, wurden ebenfalls gebeten, nach jeder erfolgten Konsultation einen Fragebogen auszufüllen. Die behandelnden Ärzte füllten die Fragebögen nach 67,9% (152/224) der durchgeführten Videokonsultationen aus. Sowohl bei den Patienten als auch bei den Ärzten überwogen Frauen (71,9% beziehungsweise 69,2%). Das Durchschnittsalter der Patienten betrug 43,2 ± 14,0 Jahre, während die teilnehmenden Ärzte 31,1 ± 3,9 Jahre alt waren. Die meisten Patienten (88,2%) und Ärzte (96,1%) bewerteten ihre eigenen Computerkenntnisse als *gut* bis *sehr gut*, und die Mehrheit in beiden Gruppen (Patienten: 93,8%, Ärzte: 98,0%) benötigte keine technische Unterstützung bei der Teilnahme an der Videokonsultation.

Die meisten Patienten nutzten Laptops oder Mobiltelefone für die Videokonsultationen, während stationäre Computer am seltensten verwendet wurden. Alle Ärzte nutzten Windows‐basierte stationäre Computer (Windows Version 10) und klinikbasierte Server.

Die demografischen Daten von Patienten und Ärzten sind in Tabelle 2 dargestellt.

**TABELLE 2:** Demografische Daten von Patienten und Ärzten.

	Patienten, n (%)	Ärzte, n (%)
Alle	128 (100%)	Evaluation von 152 Videokonsultationen (100%) durch 13 Ärzte (100%)
Männlich	36 (28,1%)	4 (30,8%)
Weiblich	92 (71,9%)	9 (69,2%)
Alter	43,2 ± 14,0 Jahre	31,1 ± 3,9 Jahre

*Unterstützung erforderlich*		
Ja	8 (6,2%)	3 (2,0%)
Nein	120 (93,8%)	149/152 (98,0%)

*Computerkenntnisse (126/128)*		
Sehr gut	53 (41,4%)	127/152 (83,6%)
Gut	60 (46,9%)	19/152 (12,5%)
Mäßig	13 (10,2%)	6/152 (3,9%)
Schlecht	2 (1,6%)	0%

*Beschäftigungsstatus*		
Angestellt	63 (49,2%)	Assistenzärzte: 8/13 Fachärzte: 3 / 13 Oberärzte: 2/13
Student/Auszubildender	18 (14,1%)	
Selbstständig	17 (13,3%)	
Beamter	11 (8,6%)	
Im Ruhestand	7 (5,5%)	
Arbeitslos	4 (3,1%)	

*Abteilung*		
Allergologie	80 (62,5%)	81 (53,3%)
Laser	24 (18,8%)	36 (23,3%)
Allgemeine Dermatologie (Ambulanz)	24 (18,8%)	35 (23,0%)

*Gerät*		
Laptop	66 (51,6%)	0
Cell phone	29 (22,7%)	0
Tablet	17 (13,3%)	0
Stationary Computer	16 (12,4%)	152 (100%)
***Betriebssystem***		
Windows	82 (64,1%)	152 (100%)
Android	27 (21,1%)	0
iOS	19 (14,8%)	0

### Patientenzufriedenheit

Die Terminvereinbarung erhielt überwiegend positives Feedback: 76 Befragte (59,4%) bewerteten sie als *sehr gut* und 36 (28,1%) als *gut*. Die Benutzerfreundlichkeit wurde bei 87 Befragten (68%) als *sehr gut* und 32 (25%) als *gut* eingestuft. Die Stabilität der Verbindung wurde von 73 Befragten (57%) als *sehr gut* und 32 (25%) als *gut bewertet*. Die Gesamterfahrung wurde von 68 Befragten (53,1%) als *sehr gut* und von 35 (27,3%) als *gut* bewertet. Insgesamt bewerteten die Patienten alle Erfahrungsdimensionen (Terminvereinbarung, Benutzerfreundlichkeit, Verbindungsstabilität und Gesamterfahrung) durchweg mit einem Median von 1 [IQR 1–2], was eine Häufung der Antworten auf dem höchsten Zufriedenheitsniveau zeigt. Die Mehrheit der Patienten (94,5%) würde die Videokonsultation weiterempfehlen. Eine Übersicht der Ergebnisse findet sich in Tabelle 3.

**TABELLE 3:** Ergebnisse der Patientenzufriedenheit.

Note (in Anlehnung an das deutsche Schulnotensystem)	Terminvereinbarung	Benutzerfreundlichkeit	Verbindungsstabilität	Gesamterfahrung
**Alle Abteilungen, n = 128**				
1 (sehr gut)	76 (59,4%)	87 (68%)	73 (57%)	68 (53,1%)
2 (gut)	36 (28,1%)	32 (25%)	32 (25%)	35 (27,3%)
3 (befriedigend)	9 (7%)	8 (6,3%)	10 (7,8%)	17 (13,3%)
4 (ausreichend)	6 (4,7%)	0%	8 (6,3%)	2 (1,6%)
5 (mangelhaft)	0%	1 (0,8%)	1 (0,8%)	3 (2,3%)
6 (ungenügend)	1 (0,8%)	0%	2 (1,6%)	2 (1,6%)
Median [IQR]	1 [1–2]	1 [1–2]	1 [1–2]	1 [1–2]
***Einzelne Abteilungen***				
Allergologie	1 [1–2]	1 [1–2]	1 [1–2]	1 [1–2]
n = 80				
Laser	1 [1–2]	1 [1–2]	1 [1–2]	1.5 [1–2]
n = 24				
Allgemeine Dermatologie	1 [1–2]	1 [1–2]	1 [1–2]	2 [1–3]
n = 24				
**Weiterempfehlungsrate der Videokonsultation**				
Ja	121 (94,5%)			
Nein	6 (4,7%)			
Enthaltung	1 (0,8%)			

Der CSQ‐Fragebogen wurde von 127/128 Patienten ausgefüllt und zeigte in allen Kategorien ein hohes Maß an Zufriedenheit. Positive Antworten (*sehr gut* und *gut*) wurden von 93,0% der Patienten für die Qualität der Behandlung, 91,5% für den Erhalt der gewünschten Hilfe, 93,0% für die Erfüllung ihrer Bedürfnisse, 92,2% für die Weiterempfehlung der Dienstleistung, 89,8% für die Zufriedenheit mit der erhaltenen Hilfe, 88,3% für die Hilfe bei der Bewältigung von Problemen, 89,1% für die allgemeine Zufriedenheit und 91,4% für die Bereitschaft, den Service erneut in Anspruch zu nehmen, vergeben. Der durchschnittliche CSQ‐Wert über alle Abteilungen hinweg lag bei 27,3 ± 5.1, was eine hohe Zufriedenheit aufzeigt. Aufgeschlüsselt nach Abteilungen erzielte die Allergologie einen Durchschnittswert von 27,5 ± 5,3, die allgemeine Dermatologie 26,6 ± 5,7 und die Laserabteilung 27,1 ± 4,0. Eine Übersicht über die CSQ‐Ergebnisse findet sich in Tabelle 4.

**TABELLE 4:** CSQ‐Ergebnisse.

CSQ‐Ergebnisse				
Items	Sehr gut	Gut	Befriedigend	Schlecht
Qualität der Behandlung^*^	53 (41,4%)	66 (51,6%)	6 (4,7%)	3 (2,3%)
Erhaltene Hilfe entsprach den Erwartungen	61 (47,7%)	56 (43,8%)	7 (5,5%)	4 (3,1%)
Ihren Bedürfnissen wurden entsprochen^*^	76 (59,4%)	43 (33,6%)	4 (3,1%)	4 (3,1%)
Weiterempfehlung an Freunde	69 (53,9%)	49 (38,3%)	5 (3,9%)	5 (3,9%)
Zufriedenheit mit der erhaltenen Hilfe	64 (50%)	51 (39,8%)	6 (4,7%)	6 (4,7%)
Half dabei, besser mit Problemen umzugehen	70 (54,7%)	43 (33,6%)	8 (6,3%)	6 (4,7%)
Gesamtzufriedenheit^*^	66 (51,6%)	48 (37,5%)	7 (5,5%)	6 (4,7%)
Bereitschaft, den Service erneut in Anspruch zu nehmen^*^	67 (52,3%)	50 (39,1%)	7 (5,5%)	3 (2,3%)

Durchschnittlicher CSQ‐Wert (alle Abteilungen)	27,3 ± 5,1 (95% KI: 26,4–28,2)			
Durchschnittlicher CSQ‐Wert (Allergologie)	27,5 ± 5,3 (95% KI: 26,3–28,7)			
Durchschnittlicher CSQ‐Wert (allgemeine Dermatologie)	26,6 ± 5,7 (95% KI: 24,3–28,9)			
Durchschnittlicher CSQ‐Wert (Laser)	27,1 ± 4,0 (95% KI: 25,5–28,7)			

*Abk*.: CSQ, Client Satisfaction Questionnaire (deutsche Version); n, Anzahl der Teilnehmer

* 127/128 Teilnehmer

In weiterführenden Analysen wurden Korrelationen zwischen der allgemeinen Patientenzufriedenheit und den Faktoren Alter, Geschlecht, Beschäftigungsstatus, Betriebssystem, verwendetem Gerät oder Abteilung untersucht. Die Korrelationsanalyse zeigte keinen signifikanten Zusammenhang zwischen dem CSQ und Alter (p = 0,737; r_s_ = 0,03). Der Mann‐Whitney‐U‐Test zeigte keinen signifikanten Unterschied in den Zufriedenheitswerten zwischen den Geschlechtergruppen (p = 0,593; U = 1718; Z = 0,53). Es gab keinen statistisch signifikanten Unterschied in den CSQ‐Werten in Bezug auf den Beschäftigungsstatus (p = 0,267; χ^2^(7) = 8,81), das verwendete Gerät (p = 0,30; χ^2^(3) = 3,69), Betriebssystem (p = 0,57; χ^2^(1) = 0,32), die Abteilung (p = 0,52; χ^2^(2) = 1,32) oder die selbstberichteten Computerkenntnisse (p = 0,80; χ^2^(3) = 1,00).

Das Alter war signifikant mit den selbstberichteten Computerkenntnisse assoziiert (p = 0,002; χ^2^(3) = 14,68), obwohl der Unterstützungsbedarf in unserer Patientenkohorte keine Assoziation mit dem Alter zeigte (p = 0,60; U = 532,5; Z = 0,52). Die Gesamtzufriedenheit zeigte eine positive Assoziation mit einer stabilen Audio‐ und Videoverbindung (p = 0,018). Interessanterweise zeigte sich kein signifikanter Zusammenhang zwischen Gesamtzufriedenheit und verwendetem Endgerät, jedoch zeigte sich eine signifikante Assoziation zwischen Benutzerfreundlichkeit und verwendetem Gerät (p = 0,011) mit der höchsten Bewertung bei Handynutzern und der geringsten bei Nutzern stationärer Computer.

### Ärztliche Zufriedenheit

Die Evaluation der ärztlichen Zufriedenheit zeigte eine positive Gesamtbewertung in den Domänen Terminvereinbarung, Benutzerfreundlichkeit und der Gesamterfahrung, während die Verbindungsstabilität und die Bewertbarkeit der Hautbefunde gemischte Schulnoten erhielt. Die Terminvereinbarung wurde von 86,2% der Befragten als *sehr gut* und von 11,8% als *gut* bewertet. Die Benutzerfreundlichkeit wurde von 86,8% als *sehr gut* und 7,9% als *gut* eingestuft. Die Verbindungsstabilität wurde von 66,4% als *sehr gut*, von 19,7% als *gut*, von 9,9% als *befriedigend* und von 2,6% als *ausreichend* bewertet. Eine kleine Anzahl bewertete sie als *mangelhaft* oder *ungenügend* (jeweils 0,7%). Insgesamt bewerteten 72,4% der Ärzte die Erfahrung als *sehr gut* und 18,4% als *gut*. Das Feedback zur Beurteilbarkeit der Hautbefunde war uneinheitlicher, obwohl dies nicht für alle Abteilungen gleichermaßen relevant war, da die Ärzte nur dann ihre Erfahrungen mit der Hautbeurteilbarkeit angeben mussten, wenn dies für ihre Konsultation von Bedeutung war. Folglich betrug die Antwortquote dieser Domäne 9,9% in der Allergologie (n = 8/81), 45,7% in der allgemeinen Dermatologie (n = 16/35) und 100% in der Laserabteilung (100%, n = 36/36). Insgesamt wurde die Hautbeurteilbarkeit von 13,3% als *sehr gut*, 13,3% als *gut*, 20% als *befriedigend*, 21,7% als *ausreichend*, 25% als *mangelhaft* und 6,7% als *ungenügend* bewertet. In dieser Domäne wurde die schlechteste Gesamtnote erzielt mit einem Median von 4 [IQR 2–5]. Die Mehrheit der Ärzte (94,7%) war bereit, die Videokonsultation für die zukünftige Nutzung weiterzuempfehlen. Eine Übersicht der Ergebnisse findet sich in Tabelle 5.

**TABELLE 5:** Zufriedenheit der Ärzte.

	Terminvereinbarung	Benutzerfreundlichkeit	Verbindungsstabilität	Gesamterfahrung	Beurteilbarkeit von Hautbefunden
**Noten aller Fachbereiche (Median, IQR und prozentuale Verteilung einzelner Noten)**					
Note *(deutsches Schulnoten‐system*	1 [1–1]	1 [1–1]	1 [1–2]	1 [1–2]	4 [2–5]
1 (sehr gut)	131 (86,2%)	132 (86,8%)	101 (66,4%)	110 (72,4%)	8 (13,3%)
2 (gut)	18 (11,8%)	12 (7,9%)	30 (19,7%)	23 (18,4%)	8 (13,3%)
3 (befriedigend)	1 (0,7%)	5 (3,3%)	15 (9,9%)	7 (4,6%)	12 (20%)
4 (ausreichend)	1 (0,7%)	3 (2,0%)	4 (2,6%)	5 (3,3%)	13 (21,7%)
5 (mangelhaft)	1 (0,7%)	0%	1 (0,7%)	2 (1,3%)	15 (25%)
6 (ungenügend)	0%	0%	1 (0,7%)	0%	4 (6,7%)
	n = 152				n = 60
***Noten einzelner Fachbereiche (Median, IQR)***					
Allergologie	1 [1–1]	1 [1–1]	1 [1–1]	1 [1–1]	3,5 [2–4,75]
	n = 81				n = 8
Laser	1 [1–1,75]	1 [1–1]	2 [1–3]	1 [1–3]	4 [3–5]
	n = 36				
Allgemeine Dermatologie	[1–1]	1 [1–1]	1 [1–1]	1 [1–1]	3 [1–5]
	n = 35				n = 16
***Weiterempfehlungsrate der Videokonsultation***					
Ja	144 (94,7%)				
Nein	8 (5,3%)				
Enthaltung	0 (0%)				

Obwohl die Gesamterfahrung der Ärzte einen Medianwert von 1 aufwies, was einer „sehr guten“ Gesamtzufriedenheit entspricht, zeigt der Interquartilsabstand der Laserabteilung [1–3] eine geringere Zufriedenheit. Dies ist in erster Linie auf die Notwendigkeit einer zuverlässigen diagnostischen Hautbefundung zurückzuführen, da therapeutische Entscheidungen in hohem Maße von der Einschätzbarkeit von Hautläsionen abhängen. Die ärztliche Gesamterfahrung und Weiterempfehlungsrate korreliert stark mit der Abteilung, in der die Videokonsultation durchgeführt wurde (*p* < 0,001). Videokonsultationen bei Patienten, die eine Laserbehandlung wünschten, erzielten im Vergleich zur allergologischen Abteilung die schlechtesten Ergebnisse in Bezug auf die Konnektivität (*p* < 0,001), die allgemeine Erfahrung (*p* < 0,001) und die Weiterempfehlung (*p* < 0,001). Darüber hinaus korrelierten Verbindungsprobleme (*p* < 0,001) mit schlechteren Noten bei der Beurteilbarkeit des Hautbefundes. Ärzte, die die Hautbefundung als schlecht bewerteten, empfahlen die Videokonsultation seltener (*p* < 0,001) und hatten eine schlechtere Gesamterfahrung (*p* < 0,001).

### Qualitative Analyse der Kommentarbereiche

Sowohl die Patienten‐ als auch die Ärztebefragung enthielt einen freien Kommentarbereich, in dem individuelle Anmerkungen gesammelt wurden. Insgesamt wurden 49 Kommentare von Patienten und 14 von Ärzten erfasst. Die im Kommentarbereich berichteten Vorteile und Beeinträchtigungen sind in Tabelle 6 zusammengefasst. Kommentare, die mehrfach erwähnt wurden, sind *kursiv* hervorgehoben.

**TABELLE 6:** Zusammenfassung der Kommentare von Patienten und Ärzten.

	Patienten 49/128 Kommentare	Ärzte 14/152 Kommentare
Audio	− *Audiobeeinträchtigung* (schlechte Qualität, Echo), Headset für Ärzte empfohlen	− Das Mikrofon des Patienten funktionierte nicht, sodass keine Kommunikation möglich war
Video	− Auflösungsprobleme (Hautbefundung nicht möglich; keine Möglichkeit, weitere Hautveränderungen zu zeigen)	− Videoverbindung war instabil oder nicht verfügbar, wenn Patienten mobiles Datenvolumen anstelle von WLAN nutzten − *Auflösungsprobleme (schlechte Video‐/Bildqualität) führten zu eingeschränkter Hautuntersuchung*
Andere	− Mangelnde Kontinuität: Unterschiedliche Ärzte führten zu einem Vertrauensverlust − *Präferenz für persönliche Gespräche* − Videokonsultationen werden als gleichwertig mit Telefonaten angesehen; kein zusätzlicher Nutzen − *Erhöhte Flexibilität bei Terminen (zum Beispiel Terminwahrnehmung während des Urlaubs oder bei Arbeit im Ausland)* − *Zeitersparnis durch weniger Anfahrt und Wartezeiten*	− Es wurden keine Zeitersparnisse festgestellt, da zeitaufwändige Nachbearbeitungsaufgaben wie die Kodierung, SAP‐Dokumentation und das Versenden von Rezepten anfielen − Die Konsultationen waren sehr zeitaufwändig, wenn Patienten mit IT‐Problemen konfrontiert waren und hierfür telefonische Unterstützung des Arztes benötigten − *Das Fehlen von Dermatoskopie und Haptik behinderte die diagnostische Einordnung* − *Videokonsultationen sind ungeeignet für die Beurteilung von Hautläsionen vor Laserbehandlungen, die Beurteilung neu aufgetretener Hautveränderungen, die Beurteilbarkeit bei Progredienz bestehender Läsionen* − *Melanozytäre Läsionen waren per Video besonders schwer zu beurteilen*

Während Patienten häufig Probleme mit der Audioqualität erwähnten, äußerten sich Ärzte häufiger zu Beeinträchtigungen bei der Videoqualität. Positive Bemerkungen über die erhöhte Flexibilität und Zeitersparnis wurden von Patienten häufiger genannt, während Ärzte negative Aspekte wie zeitaufwändige Nacharbeitung nach der Konsultation und Einschränkungen bei der diagnostischen Beurteilung erwähnten.

Da alle Ärzte identische Geräte und Betriebssysteme verwendeten (stationäre, Windows‐basierte Klinikcomputer) analysierten wir den Zusammenhang zwischen den verwendeten Geräten und Betriebssystemen bei Patienten mit der berichteten Verbindungsstabilität. Weder der Gerätetyp (p = 0,325, r_s_ = 0,088) noch das Betriebssystem (p = 0,828, r_s_ = 0,019) zeigten eine signifikante Korrelation mit der gemeldeten Verbindungsstabilität.

## DISKUSSION

Unsere Studie hatte zum Ziel, die Zufriedenheit, Akzeptanz und die wahrgenommenen Barrieren sowohl von Patienten als auch von Ärzten bei Videokonsultationen in der Dermatologie zu analysieren, um die Implementierung teledermatologischer Dienste zu verbessern. Insgesamt bietet die Teledermatologie vielfältige Möglichkeiten und kann für Erstkonsultationen, Folgetermine und die langfristige Behandlung chronischer Hauterkrankungen genutzt werden. Telemedizin fungiert auch als effektives Triage‐Instrument zur Identifikation von Patienten, die eine persönliche Betreuung benötigen.^16^, ^17^ Der Einsatz der Teledermatologie ermöglicht zudem einen besseren Zugang zu dermatologischen Behandlungen und erleichtert Patienten die Kontaktaufnahme mit Fachärzten, insbesondere in ländlichen Gebieten.^18^, ^19^, ^20^ Eine erfolgreiche Integration hängt jedoch von einer hohen Akzeptanz bei Ärzten und Patienten ab und erfordert eine gründliche Analyse potenzieller Risiken und Barrieren.

Unsere Studie liefert wertvolle Einblicke aus der täglichen Praxis und zeigt eine hohe Gesamtzufriedenheit beider Seiten, was sich in einer Weiterempfehlungsrate bei 94,5% der Patienten und 94,7% der Ärzte widerspiegelt. Diese Akzeptanz ist entscheidend für die erfolgreiche Einführung der Teledermatologie in der klinischen Praxis, da sie es Gesundheitsdienstleistern ermöglicht, einer Vielzahl von Patienten eine zeitnahe und effektive Versorgung zu bieten. Eine detaillierte Analyse ergab eine besonders hohe Patientenzufriedenheit in Bereichen wie Terminvereinbarung und Benutzerfreundlichkeit, wobei 80,4% der Befragten eine positive Gesamterfahrung berichteten. Der CSQ‐Fragebogen bestätigte ebenfalls ein hohes Maß an Zufriedenheit in allen untersuchten Dimensionen. Darüber hinaus zeigte sich eine hohe Bereitschaft zur erneuten Inanspruchnahme des telemedizinischen Dienstes, da 91,4% der Patienten angaben, diesen erneut nutzen zu wollen. Eine hohe Patientenzufriedenheit wurde auch in einer Studie von Pearlman et al. festgestellt, in der 88,9% der Befragten angaben, insgesamt mit der teledermatologischen Erfahrung zufrieden gewesen zu sein.^21^ In einer Studie von Hindelang et al. betrachteten 92% der Teilnehmer mit chronischer spontaner Urtikaria ein digitales Konzept als vielversprechende Alternative zu herkömmlichen Konsultationen und betonten dessen Bequemlichkeit und Eignung für die Behandlung chronischer Erkrankungen.^22^ In einer Studie von Ruggiero et al. wurde eine hohe Zufriedenheit unter Patienten festgestellt, die sowohl videobasierte als auch telefonbasierte teledermatologische Dienste nutzten. Patienten, die videobasierte Konsultationen in Anspruch nahmen, zeigten dabei eine höhere Zufriedenheit als jene, die ausschließlich telefonbasierte Konsultationen nutzten; der Unterschied war jedoch nicht statistisch signifikant.^23^ Eine mögliche Erklärung hierfür könnte darin liegen, dass videobasierte Konsultationen in stärkerem Maße einem persönlichen Arztbesuch ähneln als telefonische Gespräche. Ergänzende Daten aus systematischen Übersichtsarbeiten zur Implementierung der Teledermatologie berichten von einer hohen Patientenzufriedenheit (≥ 80%), die vor allem auf den erhöhten Komfort und die Verkürzung der Wartezeiten zurückgeführt wird.^24^, ^25^, ^26^, ^27^

Die Zufriedenheit der Ärzte in unserer Studie war in Bereichen wie Terminvereinbarung, Benutzerfreundlichkeit, Verbindungsstabilität und Gesamterfahrung sogar höher als die Zufriedenheit der Patienten. Diese Ergebnisse decken sich mit Daten von Mounessa et al., die eine hohe Zufriedenheit der medizinischen Leistungserbringer mit der Teledermatologie berichteten.^28^ Allerdings zeigten sich deutliche Unterschiede in der ärztlichen Zufriedenheit hinsichtlich der Beurteilbarkeit von Hautbefunden. Die Laserabteilung wurde im Vergleich zur Allergologie deutlich schlechter bewertet, was vermutlich auf die höheren Relevanz visueller Befundung vor Laserbehandlungen zurückzuführen ist, während in der Allergologie anamnestische und laborbasierte Aspekte dominieren.

Darüber hinaus zeigte unsere Studie einen klaren Zusammenhang zwischen der Verbindungsqualität, der Beurteilbarkeit von Hautbefunden und der ärztlichen Weiterempfehlungsrate der Videosprechstunde. Dies unterstreicht die entscheidende Bedeutung einer hochauflösenden Videoübertragung, einer stabilen Verbindung und zuverlässiger Geräte, insbesondere für visuell abhängige Fachgebiete.

Aus klinischer Sicht müssen die Grenzen der dermatologischen Fernbeurteilung anerkannt werden. Die fehlende Möglichkeit, Hautbefunde palpatorisch zu erfassen oder eine dermatoskopische Untersuchung durchzuführen, kann die Diagnosegenauigkeit einschränken. Daher eignet sich die Teledermatologie möglicherweise am besten für die Triage und Nachsorge, während komplexe oder unklare Fälle weiterhin eine persönliche Konsultation erfordern. In ähnlicher Weise unterstreichen die Erkenntnisse aus systematischen Übersichtsarbeiten die Bedeutung der technologischen Qualität, da eine unzureichende visuelle Beurteilung konsistent mit einer geringeren Zufriedenheit und eingeschränkten Diagnosesicherheit einherging.^24^, ^25^, ^27^ Unsere Ergebnisse müssen auch unter Berücksichtigung des Arztprofils interpretiert werden, da 60% der Teilnehmer Ärzte in Weiterbildung waren. Diese Gruppe ist zwar möglicherweise offener für digitale Innovationen, doch könnte ihre begrenzte klinische Erfahrung das Vertrauen in die Entscheidungsfindung aus der Ferne beeinflussen.

Ein hybrider Ansatz, wie von Pearlman et al. vorgeschlagen, könnte die Effektivität der Teledermatologie verbessern, solange die Videoauflösung von Digitalfotografie auf Verbraucherebene nicht mit der erforderlichen diagnostischen Qualität mithalten kann.^21^ Über die fortschrittliche Bildgebung hinaus könnte die Integration technischer Entwicklungen in telemedizinische Arbeitsabläufe wie Dermatoskopie, konfokaler Mikroskopie und künstlicher Intelligenz (zum Beispiel großer Sprachmodelle; LLM), weiteres Potenzial zur Verbesserung der Teledermatologie bieten.^29^, ^30^

Die Implementierung der Teledermatologie in bestehende Arbeitsabläufe im Gesundheitswesen birgt jedoch auch logistische Herausforderungen.^25^, ^26^, ^27^ Eine kontinuierliche Aufklärung und Schulung sowohl der Patienten als auch der Ärzte ist unerlässlich, um den effektiven Einsatz teledermatologischer Instrumente zu gewährleisten. Neben der Notwendigkeit hochwertiger Bildgebung ist es entscheidend, dass Patienten darin geschult werden, geeignete Bilder anzufertigen und Teledermatologie‐Plattformen sachgerecht zu nutzen. Gleichzeitig müssen Gesundheitsdienstleister die Kompetenz entwickeln, diese Systeme fachgerecht zu bedienen und das eingereichte Bildmaterial zuverlässig zu interpretieren. Darüber hinaus sollte die Implementierung der Teledermatologie die lokalen kulturellen Gegebenheiten sowie bestehende Ressourcenbeschränkungen berücksichtigen.^31^

Rechtliche und ethische Fragestellungen – insbesondere Datenschutz, Haftung und Plattform‐Governance – bleiben weiterhin relevant und erfordern zusätzliche legislative Maßnahmen, um eine sichere Nutzung teledermatologischer Dienste zu gewährleisten. Wirtschaftliche und qualitätsbezogene Aspekte sind ebenso bedeutsam, da die Gefahr besteht, dass der Effizienzdruck die Geschwindigkeit gegenüber der Versorgungsqualitätpriorisiert. Weitere Faktoren, die die Nutzung der Teledermatologie beeinflussen, sind die Nachhaltigkeit der Plattformkonfiguration, die digitale Kompetenz der Nutzer, ein konsistenter Internetzugang und Mobilfunkempfang, die Präferenz der Patienten für persönliche Interaktion, Bedenken hinsichtlich des Datenschutzes und der Privatsphäre sowie die Einrichtung eines tragfähigen Erstattungssystems.^31^, ^32^ Die möglichen Vor‐ und Nachteile für Patienten und Gesundheitsdienstleister, die im Zusammenhang mit Videokonsultationen diskutiert wurden, sind in Tabelle 7 zusammengefasst und bieten weitere Einblicke in die praktischen Auswirkungen der Teledermatologie in der Routineversorgung.

**TABELLE 7:** Vorteile und Nachteile von Videokonsultationen.

Vorteile für Patienten	Nachteile für Patienten
Zeit‐ und Kostenersparnis Schnellerer Zugang zu Konsultationen Zugang zu Konsultationen unter besonderen Umständen (zum Beispiel Pandemie, körperliche/geistige Einschränkungen, Geschäftsreisen) Privatsphäre und Komfort (zum Beispiel kein Verlassen des Wohnortes oder Arbeitsplatzes erforderlich) Verbesserter Zugang für ländliche Gebiete	Erstkonsultationen können schwierig sein (begrenzte Diagnosemöglichkeiten, Aufbau von Vertrauen aus der Ferne) Geeignete Geräte, technische Ausstattung und Kenntnisse erforderlich Privatsphäre/Datenschutz
**Vorteile für Gesundheitsdienstleister**	**Nachteile für Gesundheitsdienstleister**
Ressourcenschonung durch Reduzierung von persönlichen Besuchen Zeit‐ und Kostenersparnis Remote‐Arbeit möglich Geringeres Infektionsrisiko Verbesserter Zugang für ländliche Gebiete	Erstkonsultationen können schwierig sein (begrenzte Diagnosemöglichkeiten, Aufbau von Vertrauen aus der Ferne) Geeignete Geräte, technische Ausstattung und Kenntnisse erforderlich Privatsphäre/Datenschutz

Obwohl unsere Studie eine wertvolle Momentaufnahme liefert, befindet sich der Bereich der Teledermatologie in einem kontinuierlichen Wandel. Weiterführende Forschung ist erforderlich, um mit den technologischen Fortschritten, Veränderungen klinischer Arbeitsabläufe und den sich wandelnden Erwartungen der Nutzer mitzuhalten. Unsere Daten aus der Praxis zeigen eine hohe Zufriedenheit sowohl bei Patienten als auch bei Ärzten und unterstreichen die klinische Relevanz und den Nutzen von videobasierten Konsultationen in der Dermatologie. Gleichzeitig identifiziert die Studie Bereiche mit Verbesserungsbedarf, insbesondere hinsichtlich der Videoqualität und der Beurteilbarkeit von Hautbefunden. Angesichts des Fortschritts in der Teledermatologie ist eine kontinuierliche Evaluation und Anpassung entscheidend, um ihr Potenzial für Patienten sowie für das Gesundheitssystem voll auszuschöpfen. Insbesondere bestehen weiterhin Evidenzlücken, da randomisierte Implementierungsstudien, Kosten‐Nutzen‐Analysen und standardisierte Instrumente zur Messung der Zufriedenheit weitgehend fehlen.^26^

### Limitationen

Diese monozentrische Studie zu Videokonsultationen in der Dermatologie weist einige Limitationen auf, die bei der Interpretation der Ergebnisse berücksichtigt werden sollten. Die Fokussierung auf ausgewählte Teilgebiete (zum Beispiel Laserabteilung) und die geringe Stichprobengröße (128 Patienten, 13 Ärzte) schränken die allgemeine Gültigkeit und statistische Aussagekraft ein. Ein Selektionsbias könnte zu einer Überrepräsentation von Patienten geführt haben, die der Telemedizin positiv gegenüberstehen. Um die Ergebnisse zu validieren, sind weitere multizentrische Studien mit größeren und diverseren Stichproben erforderlich. Die Daten der Ärzte wurden anhand einer nicht validierten Skala erhoben. Obwohl die interne Konsistenz akzeptabel war (gemessen mit Cronbachs alpha), schränkt das Fehlen einer formalen Validierung die Interpretierbarkeit der Ergebnisse ein. Darüber hinaus wurde in der Analyse keine Clusterbildung bei ärztlichen Antworten berücksichtigt. Ethische und datentechnische Einschränkungen verhinderten die Verwendung von Mixed‐Effects‐Modellen wodurch möglicherweise Standardfehler unterschätzt und das Risiko von Typ‐I‐Fehlern erhöht wurden. Daher sollten p‐Werte mit Vorsicht interpretiert werden.

Die statistischen Analysen waren explorativ und basierten auf nichtparametrischen Methoden. Dieser Ansatz balanciert die Minimierung von falsch‐negativen Ergebnissen mit einem erhöhten Risiko für falsch‐positive Ergebnisse und unterstreicht die Notwendigkeit von konfirmatorischen Studien.

Der Studie fehlten objektive Daten zur wirtschaftlichen oder klinischen Effizienz, und zukünftige Forschungsarbeiten sollten Bewertungen der Kosteneffizienz und der langfristigen Nachhaltigkeit einbeziehen. Darüber hinaus antworteten nur 57% aller Patienten auf unsere Umfrage, wobei aufgrund fehlender Einwilligungen und Daten keine formelle Analyse aller Patienten möglich war, was die Gefahr einer Antwortverzerrung zugunsten zufriedener Patienten erhöht. Eine geringere Rücklaufquote in der allgemeinen Dermatologie, die möglicherweise durch einen höheren Anteil privater Patienten beeinflusst wurde, könnte ebenfalls die Repräsentativität beeinträchtigen.

## DANKSAGUNG

Gefördert durch das Clinician Scientist Programm der Universitätsmedizin Leipzig. Wir danken Anton Schmidt, Berlin, für die technische Unterstützung.

Open access Veröffentlichung ermöglicht und organisiert durch Projekt DEAL.

## INTERESSENKONFLIKT

Keiner.
